# Are Perceptions of Health Dependant on Social Class? Studying Soft Power and Symbolic Violence in a Health Promotion Program among Young Men at Vocational Schools

**DOI:** 10.3390/ijerph18147517

**Published:** 2021-07-15

**Authors:** Bent Egberg Mikkelsen, Frantisek Sudzina, Marek Botek, Annette Quinto Romani, Kristian Larsen

**Affiliations:** 1 Department of Geoscience & Natural Resource Management, University of Copenhagen, 1958 Frederiksberg, Denmark; 2 Department of Systems Analysis, University of Economics, 130 67 Prague, Czech Republic; sudzina@business.aau.dk; 3 Department of Materials and Production, Aalborg University, 2450 Copenhagen, Denmark; 4 Department of Economics and Management, University of Chemistry and Technology, 166 28 Prague, Czech Republic; marek.botek@vscht.cz; 5 Department of Sociology and Social Work, Aalborg University, 9220 Aalborg, Denmark; aqr@socsci.aau.dk; 6 Copenhagen University Hospitals Centre for Health Research, 2200 Copenhagen, Denmark; kristian.larsen@regionh.dk; 7 Department of Public Health, University of Copenhagen, 1123 Copenhagen, Denmark

**Keywords:** whole school approach, target intervention, symbolic violence

## Abstract

Health behaviour among young people has a social gradient, and tends to be skewed in terms of gender as well. Young men in vocational educational settings are an example where the inequality in health is apparent. Addressing this problem requires an understanding of health behaviour and its determinants in the target group in order to be able to develop interventions that can address the problem. The aim of the paper is to investigate to what extent a multicomponent intervention based on the Whole School Approach, targeting the risk behaviours, smoking, eating and physical activity that have an impact on health behaviour among male students in a disadvantaged educational setting. The paper uses self-reported longitudinal data on risk behaviours from the “Gearing up the Body” 1-year intervention program that was implemented among students at a Danish vocational school. For the analysis, we created a score model to categorise students and behaviour. Analyses suggest that interventions had only a modest impact and what evidence there is shows that the interventions reduced the health behaviour scores by 0.03 points. More specifically, we find that symbolic violence reduces the health behaviour score of the healthy types by 0.20 points, whereas soft power increases the health behaviour of the unhealthy type by 0.05 points. An explanation for the disappointing results of the “Gearing up the Body” program is tension between different understanding of what is “right” and “wrong” health behaviour. We find that the ideas of soft power and symbolic violence can contribute to a better understanding of why health and health behaviour is understood differently among vocational students. Thus, the finding demonstrates that one needs to apply a participatory approach rather than a normative approach addressing the health behaviour of disadvantaged individuals.

## 1. Introduction

The focus on unhealthy lifestyle and its negative consequences for health outcomes has increased during the last decades. Public health programs and strategies addressing the problem are being implemented at different levels in society, and by more stakeholders, and, in particular, intervention in the settings of everyday life has become a popular policy option. However, such programs and policies have an in-built risk of benefitting different social groups in an uneven manner. This has resulted in social inequalities and a split in societies, where part of the population is adopting healthy lifestyles and another part is not. This has led to a gap in lifestyles and health outcomes and has underlined that inequality in health is a serious challenge [1]. Studies has found that groups with shorter education have shorter life expectancy than other groups [2,3] and a higher risk of developing diseases such as diabetes and cardiovascular disease, compared to other groups. Individuals with a shorter education eat less fruit and vegetables than individual with higher education, who has a higher intake of fat and meat, exercise less, and smoke more [4,5,6]. Nevertheless, the inequality is not only social, but can be observed according to gender as well. Women generally eat more in accordance with the official dietary guidelines compared to men. Women eat more in accordance with nutritional recommendations, whereas men eat fewer vegetables, have a higher intake of fat and skip meals more often than women [7].

Hence, there is a need for intervention targeting the less advantaged population groups that are in the greatest need. Setting-based approaches to intervention have been showing promising results, and there is an increasing interest in reaching these groups using the settings of everyday life such as school [8]. In particular, in primary education, much work has shown that the settings approach has the ability to have an impact [9,10]. However, when it comes to secondary education, school settings tend to become more varied in terms of the socioeconomic positions of the students that are enrolled [11]. Students that enrol at vocational and other similar schools that lead to professions in non-academic fields, such as carpentry, auto mechanics, etc., have different behaviours and different health circumstances than students that are enrolled in the more academic-oriented high school system. In general, it has been found that education, socioeconomic and lifestyle factors are interrelated, favouring those with longer educations [12].

In addition, besides this challenge, schools are faced with a gap between the health behaviours of young men and young women, since females show more compliance with official recommendations for lifestyle-related behaviours [13,14]. In addition to being a societal challenge, adverse behaviour represents a problem for schools, since it tends to be linked to absenteeism and drop-outs [15]. As a consequence, vocational schools have become a preferred option for preventive strategies, where, in particular, men with shorter educations can be reached. This strategy has received considerable attention in Denmark as part of the ongoing discussion on how to increase social mobility among young people [16].

The existing empirical research on interventions targeting at-risk populations, such as students at vocational schools, is limited, and findings on interventions addressing the behaviour of students at risk during adolescence is inconclusive, with results ranging from a positive to no effect. Studies considering the impact of interventions amongst adolescents in late school stages show mixed results with predominantly no effects found [17]. Research includes studies that are based on single-component interventions in school addressing one risk factor only and multicomponent interventions that include both physical activity in school and monitoring out of school. Results from single-component interventions where the adolescents are targeted in school and with interventions targeting risk factors and behaviours [18,19,20,21,22,23] vary considerably. In these studies, results range from insignificant effects, as shown by [22], to highly significant impacts, as shown by [8]. The studies differ considerably in their design and, for instance, their durations vary from 1 day to 2 years. The literature based on multicomponent interventions in which the adolescents are targeted not only at school but also in interventions during out-of-school free time is more consistent [24,25,26]. All three of such studies used randomised controlled trials and focused on in-school activities and physical education, and involved both parents and peers in a year-long period. All three studies found a significant effect of the intervention [24,25,26].

Thus, in our approach, we claim that variation within a certain intervention type seems to arise due to the differences in intervention duration, whereas the variation between different intervention types seems to depend on whether a single or multicomponent approach is used. Overall, research using single-component interventions tends to find little impact, implying that the best way to approach adolescents’ health in school may be to use a multicomponent intervention strategy.

The traditional approach to interventions has assumed that there is no disagreement between interventionists and the subjects of the intervention regarding what the desired outcomes of the interventions should be. In other words, that there is one “right” health behaviour and that this behaviour has been defined by health experts. However, this might be an overly simplistic assumption. We speculate that not all young people agree with the advice and recommendations given by the public and that there might be a discrepancy between the goals of authorities and experts and those of the students. We also speculate that within a student population there might be different opinions on what constitutes the “right” health behaviour. The idea that multiple realities about what is right and wrong can exist, as well as the presence of a strong power dimension when it comes to health and health behaviours, can be looked upon through the analytical lenses of the concepts of symbolic violence [27] and soft power [28,29]. This provides the opportunity to understand that norms about health and health behaviours are both created and perceived by individuals, and that regulation of health and health behaviours can be achieved through more subtle and invisible pathways than we traditionally think.

Against this background, the aim of the paper is, through the analysis of pre-/post-test “Gearing Up the Body” data, to better understand why intervention impact is either not present or less than could been have expected. We perform this analysis against the conceptual framework of symbolic violence and soft power. We assume that there are more opinions on what is a “right” health behaviour and that there is a power dimension to what constitutes “right” and “wrong” health behaviour. We argue that right and wrong is, to a large degree, a matter of power and a matter of who possesses that power. We take as a point of departure that soft power can be understood as the way that authorities influence behaviour and that the resulting norms created by that power can be perceived as a form of symbolic violence by the individual. We therefore finally aim to discuss whether symbolic violence might imply that certain groups in society take on health behaviours due to normative soft power, which lead to health behaviours that these groups would not otherwise have adopted.

## 2. Conceptual Framework

The idea of using the everyday life settings such as schools as an arena for promoting better health for young people was introduced already by the World Health Organization (WHO) in 1986 through the Ottawa Charter for Health Promotion [30]. The idea has developed and matured over the past decades and has been tested with success in school setting across the world and for many different age groups [31,32]. An important insight has been that interventions needs to be anchored as an integral part of school life if change is to occur. The Whole School Approach has been an attempt to guide this kind of program implementation [33], and it underlines the importance of working at all levels of the school environment and involving all stakeholders at the school. Studies have shown that, in particular, interventions need to address not only knowledge through educational measures, but also the wider environment if they are to have an effect, especially in certain socioeconomic groups [34].

### 2.1. Whole School Approach

The Whole School Approach has developed over the past decades as a way to integrate ideas of health into such actions in a just and equitable manner in school environments. It argues that a health program needs to respect the importance of daily life settings, since health is not formed in the institutions of the health care system but in everyday life settings such as schools and workplaces [35].

Thus, the Whole School Approach to intervention development implies taking a systems approach and taking action not only within the curriculum, but across the whole school and learning environment [36]. Taking a Whole School Approach such as the “Gearing Up the Body” program (GUB) implies taking the organisational whole into account when developing and implementing the intervention. In practice, the GUB program developed to involve classroom activities in particular for the physical activity components and for health lessons integrated in the curriculum. Nonetheless, the program also addressed non-classroom activities, and in particular the eating behaviour and the canteen practices. The latter includes how meals are offered and how they are presented both in the canteen as well as in the classroom with regards to breakfast clubs. The systems approach also included the implementation of a social dimension in breakfast clubs.

### 2.2. Symbolic Violence and Soft Power

The spread of lifestyle-related disorders and diseases in modern society has demonstrated the fact that population groups respond differently to the health norms of society [37,38]. Clearly, through its policies and recommendations, society expresses what kinds of health and what kinds of behaviours are seen as beneficial and advantageous, at the same time, it therefore promotes certain behaviours as the correct ones. In this sense, society introduces certain norms, and through these, exerts power by promoting norms expressing its intentions and policy goals on how health for all can be achieved [39]. We argue that norms of “right” and “wrong” health behaviours using the concepts of soft power and symbolic violence are important when attempting to understand potential barriers and constraints faced by traditional health programs to change behaviour. Symbolic violence is closely related to the idea of soft power; however, symbolic violence as we apply the term refers to a micro-oriented approach focusing on the impact at the individual level, whereas soft power refers to a macro-oriented approach focusing on the processes at a structural level. Nevertheless, symbolic violence and soft power are interrelated through the concept of habitus, capturing the structural impact on individual behaviour.

Symbolic violence as an academic concept was originally developed to account for the individual and group-based bodily self-suppression that, for instance, lower-educated students apply to themselves [26]. For instance, we speculate that an individual might refrain from promoting certain views and opinions on health that are not in line with mainstream thought. As such, this is an influence and power that is perceived by the individual and that can lead to a certain behaviours in compliance with existing norms. This way of normative power is soft in the sense that it does not require regulation and governance—a mode of regulation behaviour that is normally referred to as hard power. Soft power as a concept explains the ability to attract and co-opt people, shaping their preferences and culture through appeal and attraction. We apply these concepts as complementary to each other, where symbolic violence refers to the micro level as perceived by the individual and soft power, on the other hand, refers to the macro level. The normative influence should be noted. It should be noted that soft power is rooted in structures, in hierarchies and in culture. As such, it can exist outside of formal institutions, and it is important to note that soft power and symbolic violence should be seen as interrelated, as part of the interplay between the individual and society.

Soft power implies that certain groups in society take on health behaviours due to a normative power; such a normative approach can affect individual perceptions of what is accepted and not accepted behaviour. Soft power implies the existence of the dominator as well as the dominated, who accept and incorporate an exchange of social values. The soft power affects the everyday life practices as individuals absorb the structures. According to Bourdieu, individuals accept a given pre-existing social order in the social system, even if they can be said to be disadvantaged by it. This “paradox of doxa”, as Bourdieu puts it, is an in-built conflict that is underlying the common sense notions which rule our social reality [40]. According to Bourdieu, symbolic violence is part of an individual’s habitus and is related to the “rules of thumb” that an individual use to guide action in relation to everyday life practices. Bourdieu claims that individuals absorb the structures and hierarchies of the social settings—the fields—in which they live and that they incorporate behavioural patterns into their “mental structures”; their habitus [41].

Symbolic violence and soft power lead to behaviours where individuals unjustly blame themselves for their own sufferings, while overlooking the role of society [42]. In the context of the dominant way of thinking about health and health behaviours, it can be speculated that these kinds of tacit and almost unconscious modes of acting under cultural and social domination, are what takes place in the case of health behaviour in vocational schools.

Symbolic violence and soft power create a discrepancy in behaviour at two levels; at an internal and an external level. The soft power at an external level implies that the students do not experience health problems to the same extent as the health authority, whereas symbolic violence at an internal level implies that the students experience a gap between their own perception of having to change and the pressure from outside of having to change [43].

As such, our conceptual foundation is founded on the assumption that “others” intentionally or unintentionally develop norms of behaviour, thus exercising a soft form of power. Furthermore, it is also founded on the assumption that the individual translates those norms about the “right” behaviour, while perceiving a symbolic form of violence, in the sense that the norms of what is the “right” health status become, through mental processes, thoughts about the self and how the self should behave in order to comply with certain norms. Central to the study is the idea that norm creation works from the outside, externally, and from the inside-out as soft power and symbolic violence, respectively [44,45].

### 2.3. The Social Cognitive Theory and Learning

We build on the ideas of Bandura from the Social Learning Theory (SLT) and Social Cognitive Theory (SCT) [44,45], in particular the idea that behaviours are shaped by observations of others and thoughts about others. Moreover, the idea that the individuals shape their own perception of whether certain behaviours are doable for the individual in practice, which is often referred to as self-efficacy. Cognitive theory argues that individual behaviours are formed by mental processes by individuals. This means that an individual takes into consideration a range of aspects, such as the perceived benefits of the action, what others think about the action, what society expects, how feasible the action is thought to be and what will happen if the action is not taken. In that sense, the mental process is a cognitive process that will include detection of what norms exist around any health aspect or health behaviour. As such, we can think about the SCT/SLT mindset as pathway or process where individuals detect and learn, and we can think about the exertion of soft power as a norm creation and communication process where influencers such as policymakers, academics, opinion leaders alike contribute to setting an agenda around a given health aspect and a given health behaviour. In other words, the norm-setters apply—intentionally or unintentionally—soft power and the translation of this ends up in something that can be perceived as symbolic violence by the individual. We will refer to this as a cognitive approach. We further build on the idea of the non-cognitive approaches, which are assumed to be working through the environment as argued for in the literature on food environments [46] and choice architectures [47]. The latter two introduce, in line with the SCT, the idea that environments have a significant impact on behaviour and that environments can be architectured, designed and engineered in order to promote certain behaviours that are thought to be healthy. Examples of choice architectures include making healthy foods more, as well as social nudges building on the idea that individuals are inspired by what others are doing [48,49], repeated exposure [50,51], salience [52,53,54], gamification [55,56].

## 3. Materials and Methods

### 3.1. Intervention Components

The “Gearing Up the Body” (GUB) intervention program was developed and implemented among students at the vocational school UCH in Holstebro, Denmark. The student target group took part in one of two different educational tracks: The Transport/Logistics and Auto Mechanics programs. The GUB program was based on a Whole School Approach [57] and consisted of four intervention components addressing students’ eating, exercise and smoking habits simultaneously. The GUB program was initiated in August 2014 and lasted for 10 months. The intervention components varied to the extent to which they were based on an individualised, collective, or structural approach (Table 1).

The “Stop Smoking” intervention component offered a course in stopping smoking to the students. The campaign addressed the negative effects of smoking though a cognitive approach that was grounded in the individualised concept of health and lifestyle. The core element of the intervention was the provision of knowledge and information. The campaign operated by displaying posters showing the negative side effects of smoking. According to the individualised approach to health promotion, this intervention was expected increase the gap in lifestyles and health, as it primarily affected the more advantaged students in the risk group, if any.

The “Breakfast Club” intervention component was aimed at counteracting students skipping breakfast though the provision of a light traditional Danish continental style breakfast served ½ h before the formal start of the teaching. This intervention suggests that behaviour can be influenced using non-cognitive approaches to food choices, operating though collective and through social learning. The core element of the intervention was social norms. The Breakfast Club, which was chaired by a teacher, takes inspiration from social interaction approaches to health promotion, and is grounded in the collective concept of health and lifestyle. Since this approach used a social environment, the intervention was expected to reduce the gap in lifestyles and health, as it affected the target group more evenly compared to an informational strategy.

The “Healthy Cafeteria” intervention aimed at changing the students’ eating habits in the cafeteria though a change in environment strategy, and was grounded in the structural concept of health and lifestyle influencing access and availability of certain foods. In structural intervention approaches, behavioural change is achieved through changes in the food environment and can be rooted in policies and strategies [58].

The Healthy Cafeteria intervention took inspiration from the nudging mindset that suggests that behaviour can be influenced using non-cognitive approaches to food choices [48]. In this case, the sugar-sweetened beverages, including energy drinks, were re-arranged, and the visual appearance was blurred, resulting in the drinks becoming harder to spot, although still available. According to the structuralised approach, this intervention was expected to reduce the gap in lifestyles and health as it affected the target group more evenly.

The “Physical Activity” (PA) component targeted the students’ exercise habits during the school day and was built into the curriculum. It attempted to change the exercise habits though a cognitive approach that was grounded in a structural approach to health and lifestyle. The intervention for the two educational tracks was constructed in two different ways. It builds on the provision in the new Danish school reform for vocational settings requiring PA to be built into the teaching, but at the same time offering a freedom of choice of approach [59]. For the Auto Mechanic program students’, the PA intervention took place once a week and lasted 90 min including team sports such as football and baseball. The Physical Activity intervention for the students in Transport/Logistics was given in the form of power breaks, which took place once a day and lasted only for 10 min including muscle exercises such as planking or lunch training. According to the structural approach, this intervention was expected this to reduce the gap in lifestyles. Table 2 presents the Whole School Approach intervention.

### 3.2. Data Collection

Data for the survey were collected using self-reports at three steps during the intervention program. The questionnaire collected data on health behaviours that were related to the different intervention components. Students were asked to fill out the survey during school hours and with the teacher as the facilitator. The first data collection was at baseline, before the intervention was implemented, in August 2014. The second data collection was at follow-up 1 in December 2014, 5 months after the start of the intervention. The third and final data collection was at follow-up 2 in May 2015, after 10 months of intervention. The data collection was based on the same survey at all three waves of data collection, using the same survey questions, which focused on eating and exercise behaviour. For instance, the survey asked questions on dietary habits such as intake of fruit and vegetables and on exercising and physical activity behaviour. We used the Danish national dietary survey model as a format. At the third wave of data collection, the questionnaire was expanded to include questions regarding the students’ general perception of their own health and their intention and willingness to engage in behavioural change. All analyses were carried out in Stata software package.

The health behaviour explored in this analysis includes physical activity, smoking and healthy diet. Physical activity data were captured by three items considering the frequency (with in the previous 12 months) of various intensity of training (respectively, low-, moderate- and high-intensity training). Considering smoking habits, we collected information about the frequency (captured per week). For the healthy diet, data consist of a cluster of items capturing students’ healthy and unhealthy eating habits, based on 15 items tapping the frequency of eating specific foods during the previous week. The items could either be reporting eating healthy diets such as oatmeal or whole bread, or unhealthy diets such as snacks and energy drinks. All variables were coded in order to capture health behaviour. Here we used “never” as “healthy” for variables such as desserts, cake, sweets, white bread, soda with sugar and energy drinks.

**Figure 1:**
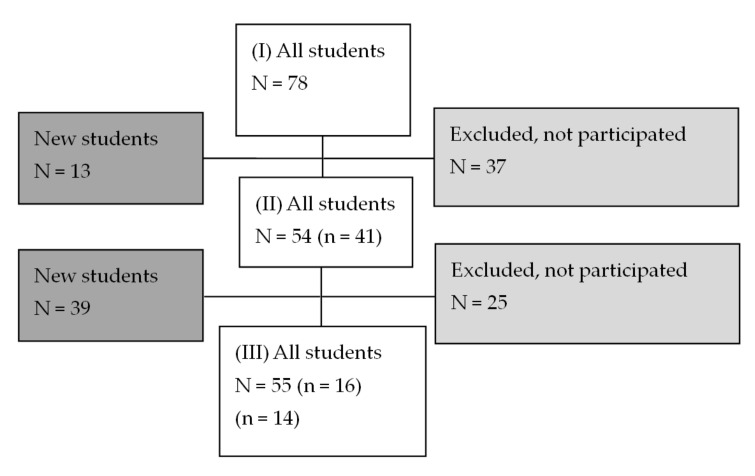
Data flow of vocational school students in the pooled and panel dataset. Note: IHC, individual health coaching. N represents all students, n represents the number of students in both waves in one vocational school in Denmark.

Figure 1 shows that in the first wave, 78 students participated in the survey; in the second wave, 41 of the students from the first wave participated and 13 new students participated; and in the third wave 16 students from the second wave and 14 from the first wave participated and 39 new students participated. We refer to the two different data sets as panel data (same student) or pooled data (any student).

The key independent variable is a dummy capturing the different waves. However, to compare the first and the final wave, we created a dummy equal to one if estimates were obtained in the final wave and zero otherwise. To explore any heterogeneous time impact, we compared the separate impact considering respective changes between the first and second wave, as well as between the second and third wave. When comparing the first and the second wave, we created a dummy equalling one if estimates were obtained in the second wave and zero if obtained in the first wave. Likewise, when comparing the second and the third wave, we created a dummy equal to one if estimates were obtained in the third wave and zero if obtained in the second wave.

The key dependent variable is a scale of health behaviour capturing eating, exercise and smoking behaviour. We used a scale, as it makes a combined variable adding eating, exercise and smoking behaviour together into one variable. We used a scale, as we assume that the health behaviour variables were correlated. Table 3 provides a detailed description of the health behavioural outcomes and a summary of statistics. The Cronbach’s alpha for health behaviour is 0.746, which includes both healthy types and unhealthy types.

In order to interpret the variables in a logical and consistent way, we standardised all variables so that they rank from 0 to 1. For example, the low intensity training is ranked from 0 to 8, which we divide by the maximum value (here 8) so we obtain a variable ranking from 0 to 1. The standardisation is carried out for all the variables, implying that we can construct the health behaviour variable.

To explore any heterogeneous impact, we divided health behaviour into the healthy type and the unhealthy type. The Cronbach’s alpha is 0.73 for the healthy type and 0.76 for the unhealthy type.

In line with the ideas of soft power and symbolic violence, we claim that this phenomenon might have heterogeneous impact on health behaviour, including both healthy and unhealthy health behaviour (see Table 3). Thus, we constructed a new variable capturing the possible influence of soft power. Here, we constructed a scale of nine items addressing students’ physical and mental health. Physical health was defined as headache, stomach pain and back pain. Mental health was defined as sad, irritable, bad mood, nervous, sleeping problem and dizzy (mental health). We argue that this captures soft power, indicating that the public health authority, which designed the interventions, has a certain understanding of reality, which they impose on vocational students, who have another understanding of reality. In other words, the public health authority is seen to hold the positions of power to “Nudge for the good” in terms of health [60,61]. Overall, it was found that the students were in fairly good physical and mental health, e.g., 23.63% of the students had a headache at least once a week, 14.54% had stomach pain, 9.09% were depressed and 20.00% were nervous. Based on these items, we first constructed a continuous variable with the value 0 to 9. Secondly, we constructed a dummy equal one if below the 50th percentile (1.45), and zero otherwise. The items were highly correlated with a Cronbach’s alpha of 0.840, validating that a scale can be constructed when adding the items. The Cronbach’s alpha could not be improved by leaving out any of the items. Constructing this new scale variable resulted in variables being ranked from 0 to 9 (see Appendix A).

To capture the variable symbolic violence we considered two separate scales which were the students’ habits that could be categorised as “need to change” or “nice to change”. Here, “need to change” is a variable captured by the questions “How do you evaluate your eating and exercise habits?” where the students’ answers can vary between very bad, bad, decent, good, and very good. In contrary, “nice to change” is captured by the question “Would you like to changes to your eating and exercise habits?” where yes is coded as one and no is coded as zero. Cronbach’s alpha for the “need to change” habits scale was 0.651, whereas for the “nice to change”, the habit scale was 0.756. Thus, there is a stronger correction related to “nice to change” compared to “need to change”. This discrepancy between the construction of the “nice to change” habits as compared to the “need to change” habits indicates the presence of symbolic violence. This indicates the power that the public health authority has imposed on the vocational students, who do not question the rules and logic that prevail within the health field. We therefore first construct a new variable capturing the difference between “need to change” and “nice to change”. Secondly, based on this continuous variable, we constructed a dummy equal to one if it is above the 50th percentile (2.84) and zero otherwise (see Appendix B).

### 3.3. Simple Differences

We use the ordinary least square (OLS) to estimate the simple differences in the intervention. The simple difference model is:

Y_ist_ − Y_ist-1_ = α + β_1_WSA_st_ + ε_ist_, (1)

This simple difference takes advantage of the longitudinal aspect of the data, which allows us to control for baseline health. Nevertheless, despite the superiority of the model taking into account baseline health, it still cannot capture any heterogeneous changes in health throughout the period that may be endogenous depending on baseline health status. However, an important concern in estimating Equation (1) is the potential endogeneity of the health behaviour implying that students are changing health behaviour depending on their health at baseline. On the one hand, it is possible that students who value health higher will be more likely to change their health behaviour. Hence, these students may be the one that benefit from the Whole School Approach. On the other hand, it is possible that the students with more unhealthy behaviours and those who have the most to gain are the ones changing their health behaviour. In both cases, a failure to account for different response during the intervention period may lead to biased estimates of the impact of the Whole School Approach. We address this issue by repeating the regressions for the healthy types and unhealthy types.

## 4. Results

Table 4 provides a description of students’ health behaviour applying the simple differences model. We present results using both the pooled and the panel data. In the panel data, we compare only students present in the baseline and the follow-up, meaning that the sample becomes very small, which makes it very difficult to obtain significant results. Hence, we attempt to proxy the changes in health behaviour by applying a pooled dataset including all students present at baseline and all students present at the follow-up.

In column (1), we compare the results from the first to the third wave and find that the interventions had no impact on the heath behaviour, and what evidence there is shows that it reduces health behaviour for the healthy types by 0.06 points. In other words, the health behaviour of the healthy types was worse after the intervention.

Comparing the heterogeneous impact of the different time periods, we find that the changes mainly took place between the first and the second wave. More specifically, the results in column (2), comparing the first and the second wave, reveal that the interventions reduced the students’ health behaviour by 0.03 points between the first and second wave. An impact that was mainly driven by a reduction in health behaviour for the healthy type by 0.05 points. In contrast, addressing changes between the second and the third wave in column (3), we find that the interventions increased the health behaviour for the unhealthy type by 0.06 points; however, the results are not significant when using the panel data.

In summary, the Whole School Approach based on the GUB program did not have the expected impact, implying a need to look for explanation and causes for this lack of impact. As mentioned earlier, the simple differences, despite their advantages, are not able to address the potential endogeneity of health behaviour, implying either that the unhealthy students, who had the most to gain, are those who did not improve their healthy behaviour or the healthy students, who care more about health, are those improving their healthy behaviour.

Another way to understand the results is to consider to what extent soft power and symbolic violence can be driving the results. Table 5 row (1) presents the impact of soft power according to our analysis. Here, the results indicate that the presence of soft power seems to increase the health behaviour of the unhealthy types by 0.05 points. Thus, the soft power embedded mainly had the intended impact on students with a low health status, indicating that soft power is a useful instrument for this subgroup. Row (2) presents the results aimed at capturing the impact of symbolic violence. Results from this analysis indicate that the presence of symbolic violence implies that the health behaviour is reduced by 0.126 score points. This impact seems to be mainly driven by a decrease in health behavioural score among the healthy types by 0.201 score points.

However, the above results indicate that soft power and symbolic violence could be an issue.

## 5. Discussions

The study investigated whether the GUB intervention program among young people at vocational school had an impact on students. The results show that the GUB program had a positive effect on some of the behavioural measures, whereas no effects were seen on the measure of health status. This is in line with other studies among adolescents at school. Most recent reviews seem to find that school interventions are effective in increasing physical activity and changing nutrition intake during school, while the result on adolescents’ weight is more dubious (for an overview see [10,62,63,64,65]).

We used the conceptual ideas of symbolic violence and soft power to examine whether these can be useful to explain the apparent lack of impact and to understand how norms of health and health behaviour are spread and how they are translated by young people in educational settings. We argue that that norms are both created as well as received—that is, there are both external and internal mechanisms. Externally, we argue that some of the pressure that the authorities exert through campaigns, recommendations and thorough interventions can be seen as examples of use of soft power, and that this power can be perceived as symbolic violence in our target group. We speculate further that pressure is translated to ideals of body image and correct behaviour among the students themselves. We argue that, in our view, the focus on a risk factor-driven health concept in combination with a “top-down” type of policy implementation may have created a “contra norm”. We argue that the contra norm comes into being when ideals and norms created in the public space are perceived as too high in the target group, and the goal set by the authorities is perceived as out of reach by the students that are targeted by intervention programs and campaigns. Here, an extreme focus on health may create a conflict within the students, implying that they feel they need to improve their health behaviour, even though they have already fairly healthy lifestyle. Thus, the use of a narrow health concept in a “top-down” perspective may increase the social stigma and self-stigma related to unhealthy behaviour without having a huge health impact.

As argued by Wyn et al. [36], there has been a tendency to conceptualise school programs as “interventions” occurring within contained systems. According to [36], the predominant focus on proving the effects of such programs has led to an increasing focus on developing evidence based on the model of randomised, clinical control trials. This narrow focus obviously tends to blur the significance of the everyday life perspective of young people. This narrow perspective neglects other factors, such as wellbeing, social connectedness and sense of coherence, which enhance or inhibit wellbeing. The studies suggest that, in order to avoid the unintended impact of interventions related to symbolic violence and soft power, it is necessary to apply a less normative and more involving approach. This can be achieved by integrating the ideas of health promotion into the wider environment of the school, including the ethos, the social, organisational and physical environment of the school. One way to do this is to include the voice of those targeted by the intervention; the young people at school, as well as the full range of other stakeholders: teachers, middle managers and top management.

From a policy perspective, it is important to address the symbolic violence in the health policy applying a broader health perspective and by addressing health intervention from a “bottom up” rather than a “top down” perspective. However, the results clearly show that the impact of the Whole School Approach seen in an overall perspective was modest. Based on these results, we suggest that it is necessary to move beyond simply exploring the pure intervention impact and to consider a broader health concept. The exploration of the soft power of traditional beliefs about what is the “right” health behaviour might be a promising way to not only increase the understanding of the inconsistency in results, but also to improve the impact of policy interventions.

Interventions targeting young people and students in educational settings have increased in popularity in a Scandinavian context compared to population-based approaches, which have proven to have a limited impact compared to their cost. In Denmark, the recent vocational school reform on physical activity is an example of such an approach where young people with low levels of PA are targeted through a regulatory approach requesting schools to provide 45 min of PA/day. Since the new bill, vocational schools have implemented this goal in different ways. In this paper we evaluated the “Gearing Up the Body” (GUB) intervention; an integrated program addressing multiple adverse health behaviours.

## 6. Conclusions

Our results indicate that when looking at the overall population as a whole, the GUB interventions had no impact on heath behaviour. However, when breaking it down into healthy and unhealthy student personas, we found that the interventions reduced the health behaviour score by 0.03 and that this impact was mainly driven by a reduction in health behaviour scores for the healthy type by 0.05. In other words, the unhealthy type seems to benefit whereas the healthy type do not. We find that soft power working as a normative pressure from outside and symbolic violence, working as the individually perceived normative pressure from the environment, might offer part of the explanation. Here we find that symbolic violence reduces the health behaviour of the healthy types by 0.20 points, whereas soft power increase the health behaviour of the unhealthy type by 0.05 points.

## Figures and Tables

**Table 1:** Intervention components. The table shows our classification of the four GUB Whole School Approach based intervention components. The left column shows the behavioural part external to the structural components and the right-hand side shows the in-built components of the school environment.

Behavioural Paradigm		Structural-Oriented Paradigm	
Smoking campaign	Breakfast club	Nudging in the cafeteria	Physical activity
Conscious	Unconscious	Conscious	Unconscious
Knowledge and information	Social norms	Accessibility and availability	Change habits

**Table 2:** Flow of the Whole School Approach intervention.

Physical Activity Once a Week					Physical Activity Twice a Week and Power Break				
August	September	October	November	December	January	February	March	April	May
2014					2015				
Breakfast									
Smoking policy									
Nudging in the cafeteria									
**Survey**			**Interview**	**Survey**				**Interview**	**Survey**

**Table 3:** Description of outcomes using an index score.

Outcome	Description	Outcome Range	Mean (Std)
**Health behaviour**	**Standardised continuous index of health behaviour. Cronbach’s alpha = 0.746**	**0–1**	**0.4675** **(0.1177)**
**Healthy type**	Standardised continuous scale capturing a healthy type. Cronbach’s alpha = 0.731	0–1	
Low-intensity training	Variable ranking from 0 if the student is “not physical active” at low-intensity level to 8 if the students are physically active at low-intensity level at least “4 h per week”	0–1	0.4659 (0.3859)
Moderate-intensity training	Variable ranking from 0 if the student is “not physically active” at moderate-intensity level to 8 if the students are physical active at moderate-intensity level at least “4 h per week”	0–1	0.4500 (0.3754)
High-intensity training	Variable ranking from 0 if the student is “not physically active” at high-intensity level to 8 if the students are physical active at high-intensity level at least “4 h per week”	0–1	0.2704 (0.3732)
Smoke often	Variable ranking from 0 if the student smokes “daily” to 6 if the student “never smoked”	0–1	0.4484 (0.4372)
Oatmeal	Variable ranking from 0 if the student never eats oatmeal to 7 if the student eats oatmeal at least two times a day	0–1	0.1400 (0.2465)
Healthy bread	Variable ranking from 0 if the student never eats healthy bread to 7 if the student eats healthy bread at least two times a day	0–1	0.4369 (0.3740)
Cereal	Variable ranking from 0 if the student never eats cereal to 7 if the student eats cereal at least two times a day	0–1	0.1428 (0.2530)
Milk	Variable ranking from 0 if the student never drinks milk to 7 if the student drinks milk at least two times a day	0–1	0.1730 (0.2439)
Salad	Variable ranking from 0 if the student never eats salad to 7 if the student eats salad at least two times a day eat	0–1	0.4909 (0.2516)
Vegetables	Variable ranking from 0 if the student never eats vegetables to 7 if the student eats vegetables at least two times a day	0–1	0.4233 (0.2489)
Fruit	Variable ranking from 0 if the student never eats vegetables to 7 if the student eats vegetables at least two times a day	0–1	0.4545 (0.3242)
Energy drink	Variable ranking from 0 if the student drinks 7 L of energy drink or more per week to 6 if the student does not drink any energy drink	0–1	0.8060 (0.2193)
Soda with suggar	Variable ranking from 0 if the student drinks 7 L of soda with suggar more per week to 6 if the student do not drink any soda with suggar	0–1	0.5787 (0.2971)
Soda light	Variable ranking from 0 if the student does not drink any soda light to 6 if the student drinks 7 L of soda light or more per week	0–1	0.1757 (0.2534)
**Unhealthy type**	Standardised continuous scale capturing an unhealthy type. Cronbach’s alpha = 0.758	0–1	
White bread	Variable ranking from 0 if the student eats white bread at least two times a day to 7 if the student never eats white bread	0–1	0.5471 (0.3288)
Dessert	Variable ranking from 0 if the student eats dessert at least two times a day to 7 if the student never eats dessert	0–1	0.7896 (0.2073)
Cake	Variable ranking from 0 if the student eats cake at least two times a day to 7 if the student never eats cake	0–1	0.7896 (0.1979)
Sweet	Variable ranking from 0 if the student eats sweets at least two times a day to 7 if the student never eats sweets	0–1	0.6909 (0.2279)
Snacks	Variable ranking from 0 if the student eats snacks at least two times a day to 7 if the student never eats snacks	0–1	0.6831 (0.2360)

**Table 4:** The impact of the interventions.

	Full Period		First Period		Second Period	
	Pooled	Panel	Pooled	Panel	Pooled	Panel
**Health behaviour**	−0.0221 (0.0238)	−0.0187 (0.0137)	−0.0110 (0.0241)	−0.0348 * (0.0187)	−0.0110 (0.0242)	−0.0094 (0.0265)
**Healthy type**	0.0235 (0.0213)	−0.0550 ** (0.0256)	0.0022 (0.0362)	−0.0475 * (0.0259)	−0.0538 (0.0369)	−0.0284 (0.0339)
**Unhealthy type**	0.0516 (0.0358)	0.0311 (0.0401)	−0.0390 (0.0269)	−0.0264 (0.0275)	0.0642 ** (0.0288)	0.0044 (0.0435)
**N**	118	26	132	82	109	32

Note: Simple differences model. Standard error in parentheses. *, ** indicate that the difference in means is statistically significant at the 0.10 and 0.05 level, respectively.

**Table 5:** The impact of soft power and symbolic violence on health behaviour score in the last and third intervention period.

	Health Behaviour	Healthy Type	Unhealthy Type
Soft power	0.0177 (0.0330)	−0.0003 (0.0494)	0.0538 * (0.0380)
Symbolic violence	−0.1257 *** (0.0295)	−0.2006 *** (0.0431)	−0.0310 (0.0381)

Note: Simple differences model. Standard error in parentheses. *, *** indicate that the difference in means is statistically significant at the 0.10 and 0.01 level respectively.

## Data Availability

Data can be obtained from the corresponding author.

## Appendix A. Description of Soft Power Using Scale

**Outcome**	**Description**	**Outcome Range**	**Mean (Std)**
**Physical and mental health**	Cronbach’s alpha = 0.840	0–9	1.4500 (1.3853)
Head ache	Dummy variable = 1 if the students had pain at least once a week, zero otherwise	0–1	0.2363 (0.4287)
Stomach pain	Dummy variable = 1 if the students had pain at least once a week, zero otherwise	0–1	0.1454 (0.3558)
Back pain	Dummy variable = 1 if the students had pain at least once a week, zero otherwise	0–1	0.2909 (0.4583)
Sad	Dummy variable = 1 if the students had pain at least once a week, zero otherwise	0–1	0.0909 (0.2901)
Irritable	Dummy variable = 1 if the students had pain at least once a week, zero otherwise	0–1	0.4000 (0.4944)
Bad mood	Dummy variable = 1 if the students had pain at least once a week, zero otherwise	0–1	0.2909 (0.4583)
Nervous	Dummy variable = 1 if the students had pain at least once a week, zero otherwise	0–1	0.2000 (0.4036)
Sleeping problems	Dummy variable = 1 if the students had pain at least once a week, zero otherwise	0–1	0.4363 (0.5005)
Dizzy	Dummy variable = 1 if the students had pain at least once a week, zero otherwise	0–1	0.0727 (0.2620)
**Soft power**	Dummy variable = 1 if the students had a physical and mental health below 1.45, zero otherwise		0.6000 (0.4944)

## Appendix B. Description of Symbolic Violence Using Scale

**Outcome**	**Description**	**Outcome Range**	**Mean (Std)**
**Need to change habits**	Cronbach’s alpha = 0.651	0–10	4.0961 (2.0317)
Eating	The answers ranks from very bad, bad, decent, good and very good.	0–4	1.8846 (1.0783)
Exercise	The answers ranks from very bad, bad, decent, good and very good.	0–4	2.200 (1.2531)
**Nice to change habits**	Cronbach’s alpha = 0.756	0–2	1.3617 (0.8450)
Eating	Dummy variable = 1 if the students are reply yes, 0 otherwise	0–1	0.2909 (0.4583)
Exercise	Dummy variable = 1 if the students are reply yes, 0 otherwise	0–1	0.2909 (0.4583)
**Symbolic violence**	Dummy variable = 1 if the discrepancy between “need to change” and “nice to change” is at or above 2.84, 0 otherwise		0.7970 (0.4038)
